# Using Specified Risk Materials-Based Peptides for Oil Sands Fluid Fine Tailings Management

**DOI:** 10.3390/ma14071582

**Published:** 2021-03-24

**Authors:** Yeling Zhu, Yuki Gong, Heather Kaminsky, Michael Chae, Paolo Mussone, David C. Bressler

**Affiliations:** 1 Department of Agricultural, Food and Nutritional Science, University of Alberta, 116 St. and 85 Ave., NW, Edmonton, AB T6G 2P5, Canada; yeling2@ualberta.ca (Y.Z.); mchae@ualberta.ca (M.C.); 2 Applied Research Centre for Oil Sands Sustainability, Northern Alberta Institute of Technology, 10210 Princess Elizabeth Ave., NW, Edmonton, AB T5G 0Y2, Canada; YUKIG@nait.ca (Y.G.); HKAMINSKY@nait.ca (H.K.); 3 Applied BioNanotechnology Industrial Research Chair, Industry Solutions, Northern Alberta Institute of Technology, 10210 Princess Elizabeth Ave., NW, Edmonton, AB T5G 0Y2, Canada; PMUSSONE@nait.ca

**Keywords:** tailings settling, biomass valorization, wastewater treatment, protein flocculant, land reclamation

## Abstract

Fluid fine tailings are produced in huge quantities by Canada’s mined oil sands industry. Due to the high colloidal stability of the contained fine solids, settling of fluid fine tailings can take hundreds of years, making the entrapped water unavailable and posing challenges to public health and the environment. This study focuses on developing value-added aggregation agents from specified risk materials (SRM), a waste protein stream from slaughterhouse industries, to achieve an improved separation of fluid fine tailings into free water and solids. Settling results using synthetic kaolinite slurries demonstrated that, though not as effective as hydrolyzed polyacrylamide, a commercial flocculant, the use of SRM-derived peptides enabled a 2-3-fold faster initial settling rate than the blank control. The pH of synthetic kaolinite tailings was observed to be slightly reduced with increasing peptides dosage in the test range (10–50 kg/ton). The experiments on diluted fluid fine tailings (as a representation of real oil sands tailings) demonstrated an optimum peptides dosage of 14 kg/ton, which resulted in a 4-fold faster initial settling rate compared to the untreated tailings. Overall, this study demonstrates the novelty and feasibility of using SRM-peptides to address intractable oil sands fluid tailings.

## 1. Introduction

Fluid fine tailings, an ultra-stable slurry primarily constituted of water and fine solids (size <44 μm), have been a major environmental concern to the oil sands industry in Alberta, Canada for several decades [1]. Due to a lack of economically-viable dewatering approaches, these intractable tailings are currently impounded in engineered tailings ponds located in the Athabasca River area. This practice raises numerous concerns regarding public health and environmental impacts, including landscape disturbance, ground water contamination, and possible dam failures with disastrous tailings release [1,2]. According to Alberta Energy Regulator [3], the total volume of oil sands fluid fine tailings has been increasing rapidly from 1075 million m^3^ in 2014 to 1253 million m^3^ in 2018. This trend is unlikely to change in the coming years [4]. Therefore, a cost-effective approach is highly desired to achieve a rapid dewatering and effective condensation of these fluid fine tailings [5].

Coagulation and flocculation are promising strategies to deal with fluid fine tailings settling and dewatering, as solid aggregates with enlarged sizes have reduced stability when subject to gravimetric settling [1,5]. In essence, coagulation reduces repulsions between solid particles, generally through adjusting pH or using chemicals such as salts (salt coagulants that are readily to be hydrolyzed in use, or that have been pre-hydrolyzed) or low molecular weight charged polymers, which reduce the net surface charges of the solids. In contrast, flocculation involves the formation of bridges between solid particles, typically triggered by high molecular weight polymers [6].

Though certain coagulants and flocculants have been piloted or commercialized for fluid fine tailings treatment, none of these products have yet to meet industrial expectations, mainly due to unfavorable side effects on downstream processing strategies and/or environmental impacts. For instance, a common coagulation approach is the composite/consolidated tailings (CT) process, in which fluid fine tailings pretreated with a coagulant (i.e., gypsum) are mixed with the underflow of fresh tailings (rich in coarser solids such as crushed rocks and sands) to generate a non-segregating sediment that ends with less than 40 wt.% water (Masliyah et al., 2010). However, poor reliability of tailings condensation was observed in several pilot tests because of the complexity and difficulty in actual operation [3]. For flocculation techniques, current inroads have been made primarily on developing synthetic polymer-based flocculants, e.g., polyacrylamide (PAM), polyethylene glycol (PEO), and their derivatives with modified chemical composition, molecular weight, and molecule structure (linear, block-copolymer, or branched) [7]. Nevertheless, as these polymers are essentially derived from petrochemicals, implementing them increases the demand for non-renewable petroleum resources.

Developing novel coagulants/flocculants from various biomass resources, including protein/peptides [8,9], cellulose [10], and chitin/chitosan [11], has recently drawn extensive attention for the treatment of various tailings streams due to the feedstock’s wide availability and low cost [12]. In contrast to conventional aggregation agents that incur unfavorable environmental footprints, the nontoxicity and potential biodegradation of biomass-derived materials make them more suitable for applications in large-scale open-air tailings management [13]. According to previous research on the interaction between suspended particles and aggregation agents [13,14], the mechanism governing how peptide materials enable tailings settling can be inferred as following. At lower pH regimes in which peptides are positively charged, the cations may serve as a charge neutralizer for solid particle surfaces that facilitates particle-particle adsorption by reducing the electrostatic repulsion; in near neutral or alkaline conditions, the negatively charged peptides, probably with the assistance of certain coagulant (such as gypsum), could function as a flocculating agent to bridge solid particles. In addition, once tailings separation is complete and the supernatant is removed, the proteinaceous coagulants/flocculants retained in the sediment may greatly benefit landscape reclamation by providing essential nutrition for plant growth, which may be of great interest to industrial oil sands operators.

Specified risk materials (SRM) refer to a group of animal tissues from slaughterhouse industries such as brain, trigeminal ganglia, eyes, tonsils, spinal cord, and dorsal root ganglia of cattle over 30 months age [15]. Compared to other tissues, these proteinaceous materials have a greater likelihood to carry bovine spongiform encephalopathy, a serious animal disease. As the Canadian Food Inspection Agency (CFIA) banned SRM from all human food, animal feed, and fertilizer purposes, the majority of the SRM production in Canada (~300,000 tons per year) is currently confined to landfilling or incineration at an average cost estimated to be around 207 USD/ton [16]. Thermal and alkaline hydrolysis creates an opportunity to valorize SRM by converting them into safe-to-use peptides, which can be used for numerous value-added applications [17,18].

This study provides the first comprehensive evaluation of using SRM-peptides as potential aggregation agents for oil sands fluid fine tailings management. Specifically, this article examines the materials’ performance in the phase separation of two fine tailings streams, i.e., synthetic kaolinite tailings (SKTs) and industrial fluid fine tailings (FFTs). Two subprocesses in tailings phase separation, i.e., tailings settling and sediment condensation, will be assessed to provide insights into the mechanism by which SRM-derived peptides serve as tailings settling agents.

## 2. Materials and Methods

### 2.1. Chemicals and Materials

Rendered SRM were provided by a large rendering company and kept in a sealed bucket at −20 °C before use. Flopam A3338, a commercial hydrolyzed-PAM (HPAM) purchased from SNF Canada Ltd. (Edmonton, AB, Canada), was used as an industrial control. Synthetic process water (SPW), used for tailings preparation and diluent for FFTs, was prepared by completely dissolving the following salts in distilled water: 28.3 parts per million in weight (ppm) KCl, 545 ppm NaCl, 39.3 ppm MgCl_2_, 41.5 ppm CaCl_2_, 237 ppm CaSO_4_, 895 ppm NaHCO_3_, and 443 ppm Na_2_SO_4_. The pH of SPW was preset to three different levels (i.e., 8, 8.5, and 9) by pipetting 10 wt.% hydrochloric acid or 10 wt.% sodium hydroxide aqueous solution, to match the typical pH range of real oil sands tailings. PolyGloss-90 kaolinite, purchased from Kamin LLC (Macon, GA, USA) was used due to its similar specific surface area (22 m^2^/g) to real oil sands kaolinite. SKTs were prepared by dispersing 4 wt.% PolyGloss-90 kaolinite in SPW (three pH levels being 8, 8.5, and 9), followed by mechanical agitation with an overhead mixer (Model 7790-751B, Phipps & Bird Inc., Richmond, VA, USA) for 30 min and stationary hydration for 1 h, before being used as the model tailings; the three pH levels of the SKTs were found to be 7.8, 8.2, and 8.4. As-received industrial FFTs, containing 24.7 wt.% solids and 1.2 wt.% bitumen, were directly collected from an Athabasca oil sands tailings pond. The ionic strengths of the original FFTs, measured by an independent lab, are listed here: 306 ppm Na^+^, 23 ppm K^+^, 51 ppm Ca^2+^, 19 ppm Mg^2+^, 138 ppm Cl^-^, and 393 ppm HCO_3−_; no CO_3_^2−^. Following a standard protocol, the original FFTs were mixed with SPW to obtain a diluted FFTs stream (termed as d-FFTs hereafter) with a final solids content of 8.9 wt.% and pH of 8.2, which was used as the industrial tailings in this study.

### 2.2. SRM Hydrolysis and Peptides Recovery

Rendered SRM was treated in a thermal hydrolysis procedure based on a Canadian Food Inspection Agency certified protocol for safe handling and disinfection [19]. Specifically, 500 g of rendered SRM premixed with 500 g Milli-Q water were transferred to an enclosed pressure vessel, followed by a thermostatic process of 180 °C and pressure of 174 psi for 40 min; this mixture was agitated at a constant rate of 200 rpm by a mixing paddle throughout the hydrolysis. After filtering out bone debris from the post-hydrolysis mixture using 0.45 μm filter paper, the obtained peptide solution was spray dried and then stored in a sealed container at 4 °C before use.

### 2.3. Characterization of SRM-Peptides, SKTs, and FFTs

SRM-peptides were characterized using tricine-sodium dodecyl sulfate-polyacrylamide gel electrophoresis (tricine-SDS-PAGE) following a method by Schägger [20]. In brief, the SRM sample was prepared by denaturing 0.1 wt% SRM-peptides overnight in a reducing buffer that contained 4 wt% SDS, 2 vol% 2-mercaptoethanol, 10 wt% glycerol, 0.016% Coomassie Blue G-250, and 50 mM Tris/HCl. The denatured sample (10 μm), as well as standard protein markers in the range of 10–250 kDa, was then loaded to a Mini-Protean TGX Precast Protein Gel, and the electrophoresis was carried out using a Mini-Protean II electrophoresis unit (all from Bio-Rad Laboratories, Inc., Hercules, CA, USA). After the fractionation, the proteinaceous materials in the gel were visualized using a Pierce Silver Staining Kit (Life Technologies, Inc., Folsom, CA, USA).

SKTs and d-FFTs, two tailings streams used in this study as sample tailings, were subject to cation exchange capacity and particle size distribution analyses. SKTs were directly used as samples for these two analyses, while standard Dean-Stark extraction with toluene as refluxing solvent was applied for d-FFTs to obtain the contained solids for sample preparation. Cation exchange capacity measurement was carried out following a previous methodology [21], and the results are presented in the form of methylene blue index; particle size distribution analysis was conducted using a laser scattering size analyzer (LA-950, Horiba Ltd., Kyoto, Japan) following a standard industrial protocol [22].

### 2.4. Cylinder Settling Test

To investigate tailings settling behaviors, 250 g of SKTs (three pH levels) were transferred to each graduated cylinder (250 mL) followed by addition of aggregation agents. Specifically, HPAM was added at the optimum dosage for this type of tailings (i.e., 160 g per ton of kaolinite clays) in the form of 0.02 wt% aqueous solution, while SRM-peptides were added as dry powders at three serial dosages labelled as L1-L3: 10, 30, and 50 kg per ton of kaolinite clays, respectively.

For the case of d-FFTs settling (one pH level), all the steps are the same except that the optimum dosage of HPAM for this type of tailings was slightly higher (i.e., 180 g per ton of clay solids), and SRM-peptides were added at five serial dosages from L1 to L5: 4, 14, 22, 29, and 49 kg per ton of clay solids in the same order. After plunging at a rate of 1 plunge/s for 20 s to completely disperse the aggregation agents, the cylinders were left untouched, with the mudline height being recorded over a 48-h period. To quantify the ultimate phase separation efficiency, the final volume of the sediment and supernatant were recorded. The supernatant pH at 2 h was measured using a pH meter (AG SevenCompact^TM^ pH/Ion S220, Mettler Toledo, Schwerzenbach, Switzerland). The total mass of the suspended solids after 48 h was quantified by filtering the final supernatant through a Whatman membrane filter paper (pore size of 0.45 µm), drying the filter paper, and then recording the mass change.

### 2.5. Statistical Analysis

All experiments were conducted with at least 3 replicates (unless otherwise specified) and results were presented in the form of mean value ± standard deviation. The statistical analyses of the data were carried out using Minitab (version 15; State College, PA, USA), a statistical software package. Single factor Analysis of Variance (ANOVA) was applied to the data populations examined to identify significant differences among mean values, based on the Least Significant Difference (LSD) criteria with a 95% confidence level.

## 3. Results and Discussion

### 3.1. Characterization of SRM-Peptides and Tailings Samples

#### 3.1.1. SRM-Peptides

**Figure 1:**
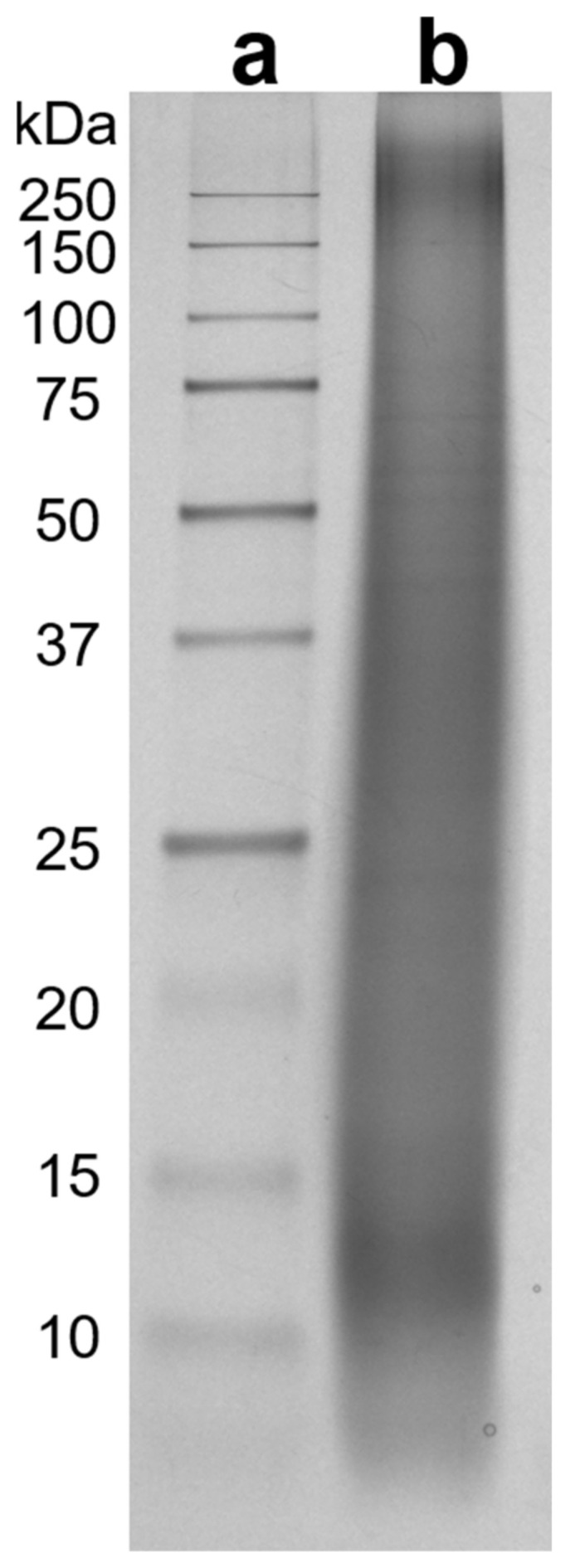
SDS-PAGE of (**a**) standard protein markers and (**b**) 0.1 wt.% specified risk materials (SRM)-peptides.

The aggregation of different suspended solids by polymer materials is highly dependent on their molecular weights; it has been extensively identified that polymer materials with a magnitude of size closer or higher than that of the suspended solids exhibit greater efficiency [13,14]. The molecular weight of SRM-peptides was analyzed using SDS-PAGE. As shown in Figure 1, SRM-peptides had a wide molecular weight distribution, with concentrated regions at around 10–50 kDa and 250 kDa. In contrast to unhydrolyzed feedstock that had molecular weight mostly above 250 kDa [19], peptides in the SRM-peptides exhibited a significant reduction in molecular size, consistently with a previous report that employed size-exclusion liquid chromatography analysis [23]. The apparent bands at ~12–14 kDa could be attributed to hemoglobin subunits that have been reported to exist in meat and bone meals [23], while the bands at 250 kDa may correspond to the more recalcitrant fractions that were not efficiently hydrolyzed. Since water-soluble linear polymers or oligomers tend to exhibit a random coil configuration in diluted aqueous solution, the root mean-square (RMS) end-to-end radius, ${\vec{R}}^{2}{}^{\frac{1}{2}}$, an estimate of the time-averaged molecule’s size, can be predicted based on the molecular weight information [24]. For instance, using Formula (1), the ${\vec{R}}^{2}{}^{\frac{1}{2}}$ of a common non-ionic linear molecule with molecular weight of 250 kDa is around 30 nm. Considering the negatively charged nature of peptides at a pH above 8 [19], it is expected that their effective size could be slightly larger than the non-ionic case (i.e., ~30 nm), due to the electrostatic repulsion between charged segments, Equation (1):

(1) $$\langle {\vec{R}}^{2}\rangle {}^{\frac{1}{2}}\;\left(nm\right)=0.06\times {\left(molecular\;weight\;in\;Da\right)}^{\frac{1}{2}},$$

#### 3.1.2. Tailings Samples

**Figure 2:**
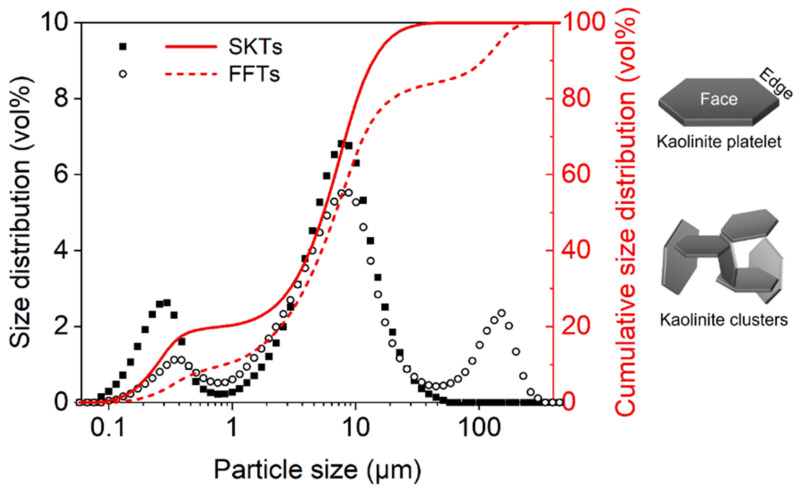
Particle Size distributions of synthetic kaolinite tailings (SKTs) and industrial fluid fine tailings (FFTs). The schematics at the right show the structures of kaolinite platelet and kaolinite clusters that potentially correspond to the 1st and 2nd peaks (starting from left) of SKTs, respectively.

The size information of solid particles of SKTs and FFTs is presented in the form of particle size distribution. As indicated in Figure 2, the size of kaolinite particles in SKTs shows a bimodal distribution, which is common for kaolinite suspensions at near neutral pH [25,26]. The primary peak at ~0.3 μm (or ~300 nm) is comparable to the length of a single kaolinite platelet and was most likely contributed by the suspended kaolinite platelet in the slurry, as schematized by the left inlet in Figure 2; in comparison, the stronger secondary peak at 6–10 μm was probably due to the presence of kaolinite clusters (also termed “flocculi”), which have a size typically in the range of 10–30 μm. According to previous research [25,27], these kaolinite clusters (the right inlet in Figure 2) can be considered as kaolinite platelets-constructed scaffolds that are joined together in the thermodynamically favored face-to-edge and edge-to-edge configurations and therefore exhibit certain structural robustness even at high turbulent shear rate during laser scattering size analysis.

In contrast to SKTs, the particle size distribution of industrial FFTs gives a trimodal distribution with local maxima at ~0.4 μm, 6–10 μm, and 100–200 μm, as shown by the hollow circles and dashed line in Figure 2. The former two peaks can be attributed to the presence of kaolinite and illite, as kaolinite is generally the most abundant clay minerals in oil sands FFTs followed by illite and mica [6]. The third peak may be correlated to the less abundant minerals such as quartz particles [28]; though not considered as fine solids in the oil sands industry (size <44 μm), the large solids constituting the third peak were found to make up ~20 vol% of the total solids in the aged FFTs, probably due to the colloidal stabilization effect of the surrounding clays [27]. Therefore, it is likely that these large solids may readily settle down once the problematic suspended clays (primarily kaolinite) are removed.

Taken together, the sizes of solid particles in tailings were found to vary remarkedly from several hundreds of nm to tens of μm. Considering the SRM-based peptides have an estimated effective size of up to ~30 nm, it is expected that the peptides, especially the fractions with larger molecular sizes, may serve as flocculating agents for bridging the target solid particles.

The methylene blue index of the two tailing streams was also determined to assess the cation exchange capacities of the fine solids in tailings. The methylene blue index of SKTs was found to be 4.1 meq/100 g, which matches the common range (3–5 meq/100 g) for pure kaolinite [29]. In contrast, FFTs were found to carry a higher methylene blue index of 14.1 meq/100 g, which is reasonable due to the potential presence of certain non-kaolinite clays in actual oil sands fluid tailings, such as illite (typically 10–40 meq/100 g) and montmorillonite (~100 meq/100 g) [29].

### 3.2. Using SRM-Peptides in Treating Synthetic Kaolinite Tailings (SKTs)

To investigate the effect of the SRM peptides on the phase separation of tailings, SKTs were incorporated into a cylinder settling test, to provide a baseline for the more complicated FFTs. Since a mudline between the solid-lean supernatant and the solid-rich sediment can be clearly observed in all settling tests, the movement of mudline, presented in the form of normalized mudline height (height of sediment divided by the original height of tailings) over time, is used in this study to evaluate the phase separation of sample tailing. Based on tailings phase separation, an in-depth understanding of the effects of SRM-peptides on a series of settling parameters, including initial settling rate, sediment condensation, and supernatant pH, are discussed in the following subsections.

**Figure 3:**
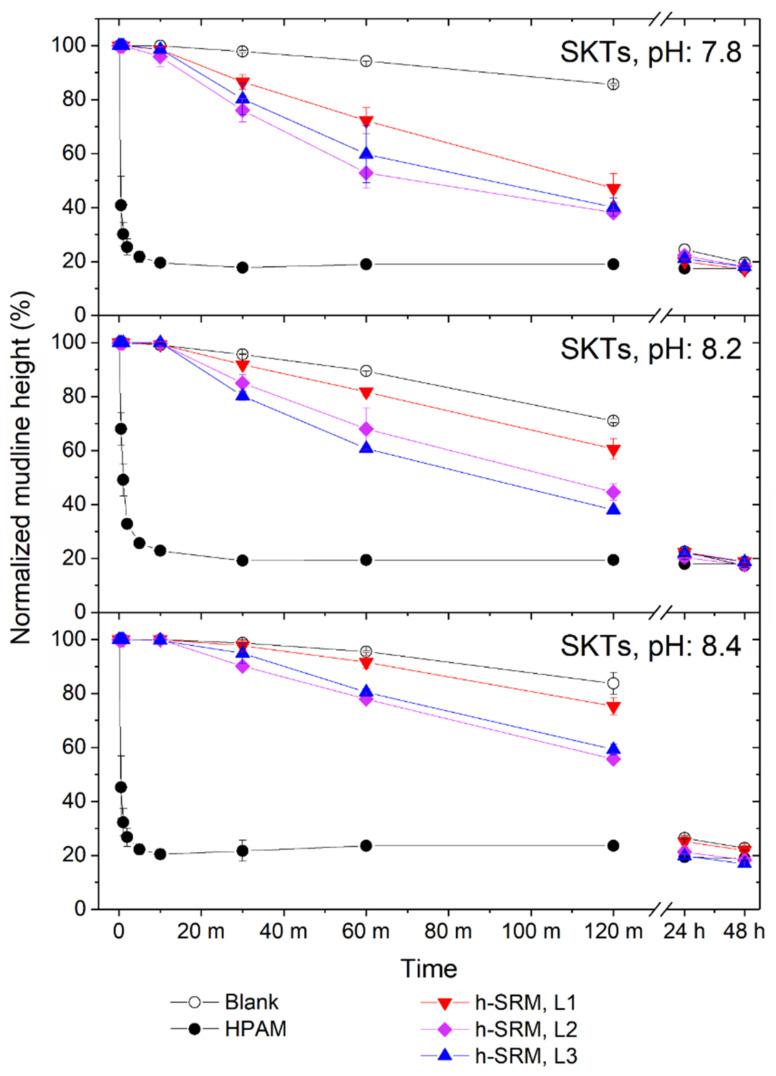
Mudline descending of SKTs, presented in form of Normalized mudline height over Time, with different initial pH: 7.8, 8.2, and 8.4 (from top to bottom). Tailings were treated with specified dosage of SRM-peptides. The blank control and the industrial control (HPAM; optimum dosage) were tested in parallel for comparison. The units “m” and “h” at the Time-axis correspond to min and hour, respectively; h-SRM refers to SRM-peptides at three dosage levels (L1–L3) from 10 kg to up to 30 kg per ton of kaolinite solids. Error bars represent the standard deviation of each experimental triplicate.

SKTs settling is presented in the form of mudline height descending over time, as shown in Figure 3. A 2-stage phase separation was clearly observed for the settling of SKTs (4 wt.% kaolinite suspensions), regardless of the different pH of the SPW used for tailings preparation. Specifically, all SKTs samples were found to settle linearly within the initial 2 h, except the cases of HPAM where linear settlings were complete within the initial 1 min. After that point, the mudlines continued to descend but at a much lower speed. According to the previous work reviewed by Wang et al. [5], such 2-stage phase separation of SKTs can be attributed to a typical settling-condensation process (or settling-consolidation process). Though not clearly distinguished in certain cases, the transition between the two stages is closely associated with the behaviors of fine solid movement in tailings slurry. Specifically, in the settling stage where the tailings suspension is diluted, the mudline generally descends at a constant rate, as the movement of a solid particle primarily depends on the characteristics of the particle itself, as well as its interaction with the surrounding fluid; in the condensation stage where the bottom sediment accumulates more solids, mudline descending performs at a much reduced rate, as the hindrance from other nearby solid particles becomes dominating [5,30]. Therefore, the SKTs phase separation behaviors in the 2 stages are evaluated separately as follows.

#### 3.2.1. Tailings Settling and Sediment Condensation

**Figure 4:**
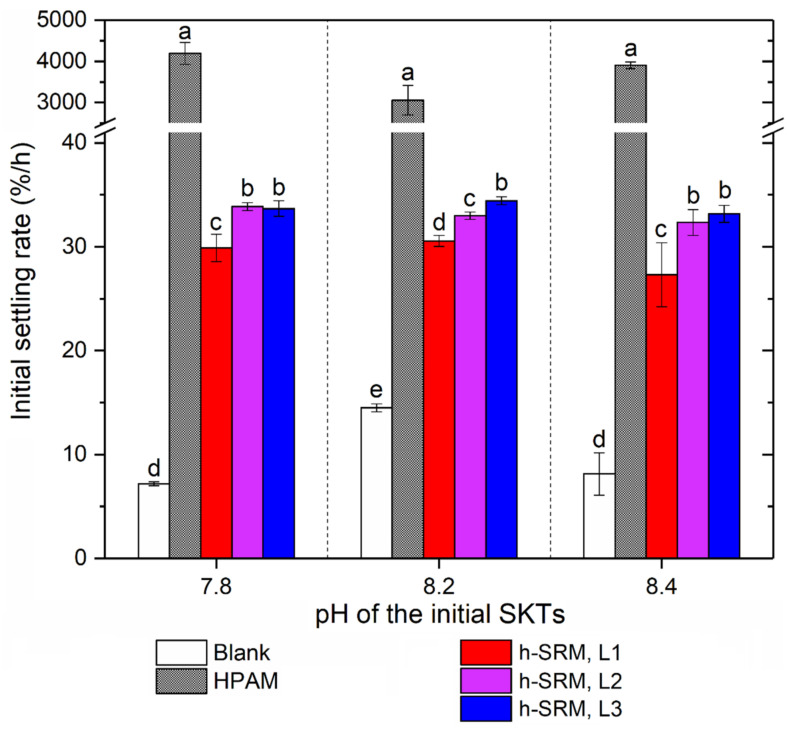
Time-average Initial settling rate ($\bar{R_{i}}$) of SKTs. SRM-peptides are labelled as h-SRM, added at three dosage levels (L1–L3) from 10 kg to up to 30 kg per ton of kaolinite solids. Error bars represent the standard deviations of experimental triplicates. Within a given pH group, bars that are annotated with the same letter (**a**–**e**) are statistically similar at a 95% confidence level.

For the settling stage, the phase separation is characterized in the form of time-average initial settling rates, $\bar{R_{i}}$, which are calculated over the initial linear mudline descending range. The time length of this linear range was found to be 1 min for HPAM and 2 h for all other groups (Figure 3). As shown in Figure 4, the untreated SKTs (blank controls) gave the lowest $\bar{R_{i}}$ in the range of 7–15%/h, which again supports the ultra-stable nature of kaolinite suspensions [5]. The incorporation of HPAM was found to substantially boost the $\bar{R_{i}}$ by 2 magnitudes (Figure 4), regardless of the initial tailings pH, which can be correlated to the flocculation effect of HPAM on forming inter-particle bridging between kaolinite particles. For all experiments using SRM-peptides, the settling rate was found to be ~2–3 fold higher than that of the blank control, suggesting that adding SRM-peptides speeded up tailings phase separation, though such improvement was not as big as HPAM. Such improvement in $\bar{R_{i}}$ was primarily contributed by the protein/peptide fractions (~89 wt%) in SRM-peptides, as the other molecules (primarily lipids) are unlikely to serve as flocculant [13]. Particularly, $\bar{R_{i}}$ appeared to initially increase and then plateau with an increase in the dosage of SRM-peptides from 10 to 50 kg/ton, which could be attributed to the saturated adsorption of peptides at kaolinite surfaces. In addition, it was also noticed that at the starting of tailings settling for all the cases except HPAM, there was a residence time (~10 min) when no obvious settling took place (Figure 3). Such residence time can likely be attributed to the relaxation time required for the SRM-peptides (added in dry) to hydrate in tailings and then serve as aggregation agents. To eliminate such relaxation time in practical application, SRM-peptides may be prepared in solution prior to use.

The condensation stage of SKTs starts at around 10 min for HPAM as the mudline descending rate was observed to substantially decrease afterwards (Figure 3). When SRM-peptides were used, the start of the condensation stage of SKTs could not be clearly observed, though it must have occurred at some point between the 2 and 24 h timepoints. In contrast to the huge variation in the settling stage, the mudline height of SKTs at 48 h appeared to be only subtly influenced by the use of HPAM or SRM-peptides. In addition, the total content of suspended solids (>45 μm) in all the final supernatants (48 h) was found to be less than 0.02 wt.% of the total released supernatant, suggesting a superb solid removal efficiency from bulky slurry in all groups. Overall, the use of SRM-peptides improves the initial settling of SKTs, while the extent of sediment condensation is barely affected.

#### 3.2.2. Effect on Supernatant pH

**Figure 5:**
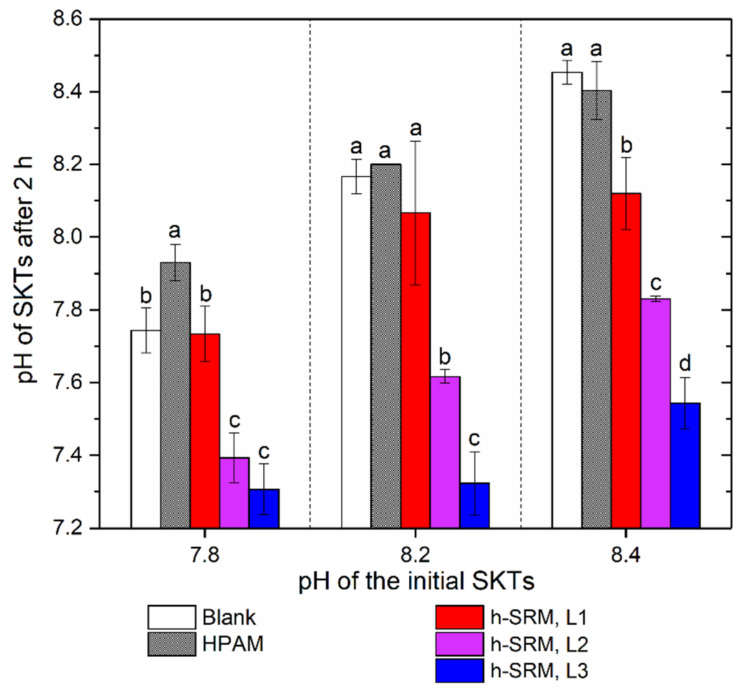
Supernatant pH of SKTs at 2 h of tailings settling. SRM-peptides are labelled as h-SRM, added at three dosage levels (L1–L3) from 10 kg to up to 30 kg per ton of kaolinite solids. The semi-bar represents the standard deviation of each experimental triplicate. Within a given pH group, bars that are annotated with the same letter (**a**–**d**) are statistically similar at a 95% confidence level.

In the mined oil sands industry, an average between 80–90% of the total water needed for oil sands extraction comes from the recycled water (supernatant) in tailings ponds [31]. As the optimum pH of water for oil sands processing typically falls between a range of 8.5–9, it is vital to understand the effect of SRM-peptides on supernatant pH before it can be used for industrial application. The supernatant pH of SKTs at 2 h of tailings settling was recorded with the results summarized in Figure 5. Increasing the dosage of SRM-peptides was found to correlate with a reduced supernatant pH compared to the blank controls; however, even in the most extreme case observed where the supernatant pH was reduced from 8.5 to 7.5 (50 kg/ton SRM-peptides), the pH variation was still within the typical range for the oil sands process water. The reason for such pH reduction could be the introduction of acidic materials such as the SRM-based peptides, which possibly carry carboxylic acid groups. The acidic compounds generated through peptide degradation, which have been identified as an important factor for pH change [32], may play a less important role in this study, as the elapsed time (2 h) would likely be too short to facilitate generation of products that could change the pH, particularly in a buffered system.

### 3.3. Using SRM-Peptides in Treating Diluted Industrial Fluid Fine Tailings (d-FFTs)

**Figure 6:**
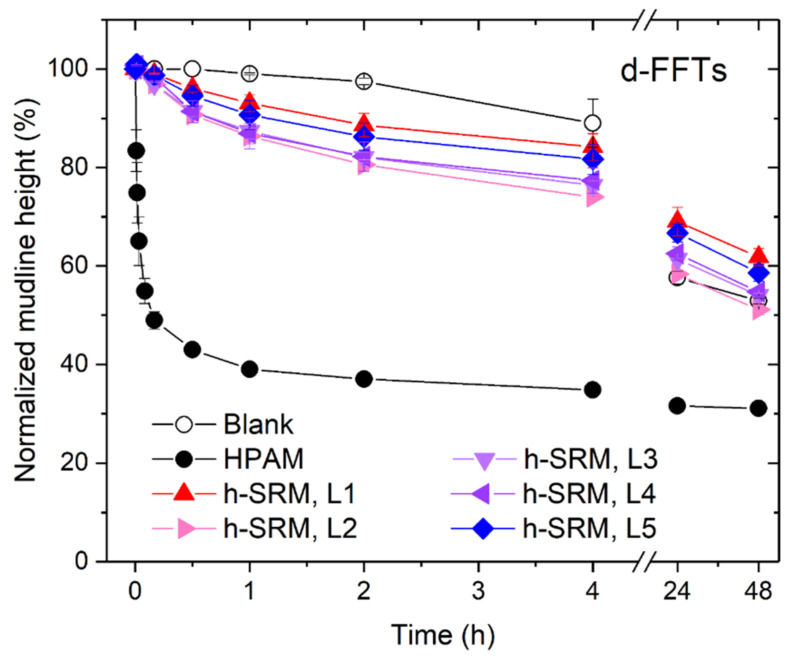
Mudline descending of d-FFTs, presented in the form of Normalized mudline height over Time. SRM-peptides are labelled as h-SRM, added at five dosage levels (L1–L5) being 4, 14, 22, 29, and 49 kg per ton of solids. Semi-bar represents the standard deviation of each experimental triplicate. The blank control and the industrial control (optimum dosage) were tested in parallel for comparison. Error bars represent the standard deviation of each experimental triplicate.

**Figure 7:**
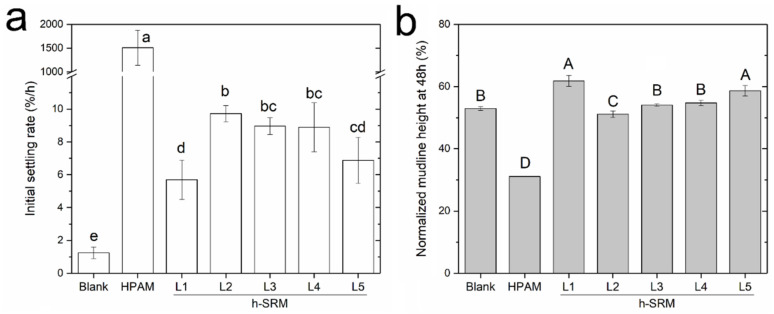
(**a**) Time-average Initial settling rate ($\bar{R_{i}}$) and (**b**) Normalized mudline height at 48 h of d-FFTs settling test. SRM-peptides are labelled as h-SRM, added at five dosage levels (L1–L5) being 4, 14, 22, 29, and 49 kg per ton of solids. Semi-bar represents the standard deviation of each experimental triplicate. Within each panel, bars that are annotated with the same letter (**a**–**d** in panel **a**; **A**–**D** in panel **b**) are statistically similar at a 95% confidence level.

In addition to model kaolinite tailings, the produced SRM-peptides were tested on diluted industrial fluid tailings streams (i.e., d-FFTs). As summarized in Figure 6, the mudline descending of d-FFTs also showed a 2-stage phase separation that is similar to the model kaolinite tailings. For the settling stage, $\bar{R_{i}}$ are calculated over the duration of linear mudline descending, as aforementioned, and the results were provided in Figure 7. Compared with the model kaolinite tailings (SKTs), the $\bar{R_{i}}$ of d-FFTs with the use of different aggregation agents followed a similar trend of HPAM > SRM-peptides > blank control. It was also noticed that d-FFTs settled at a much slower rate than SKTs. For instance, the untreated tailings were found to settle with a $\bar{R_{i}}$ of 1.3 ± 0.3%/h, much lower than the blank controls of SKTs ($\bar{R_{i}}$: 7.2–14.5%/h), which could be attributed to the complex composition of d-FFTs, such as the presence of intractable montmorillonite and bitumen-induced viscosity increase [33]. On the other hand, HPAM improved sediment condensation by reducing the final normalized sediment height of d-FFTs at 72 h from ~50% (blank control) to ~30%, as shown in Figure 6 and Figure 7. Such reduction in sediment height suggested a greater compactness of sediment that is favored in terms of producing stackable tailings. More importantly, the total concentration of suspended solids (>0.45 μm) in all the final supernatants (48 h) was found to be less than 0.01 wt.% for all peptide-treated groups. This suggests that it may be possible to recycle the water directly for continuous oil sands extraction operation.

In contrast, the effect of SRM-peptides on d-FFTs was found to be more complex. Both $\bar{R_{i}}$ and the final mudline height were found to exhibit a Gaussian (or monomodal) distribution over the test range of SRM-peptides dosage. Specifically, increasing SRM-peptides was found to first increase $\bar{R_{i}}$ and then decrease, with the final mudline heights displaying an opposite trend. Of all the dosages of SRM-peptides examined, 14 kg/ton was found to be the optimum for tailings settling, with $\bar{R_{i}}$ reaching as high as ~4 folds of the blank control, but with a comparable final water release. One explanation for the Gaussian performance of $\bar{R_{i}}$ is the competitive adsorption of low-MW and high-MW peptides at kaolinite surfaces. As elaborated by Gregory and Barany [14], competitive adsorption may take place when a secondary adsorbate is introduced to a solid-adsorbate system and partially substitute the primary adsorbate. In this study, since both clay surfaces and peptides were negatively charged under the pH employed (~8.2), attachment between these two can be achieved through the deposited multivalent cations at clay surfaces (such as Ca^2+^) [1,34]. Thus, increasing SRM-peptides dosage could trigger more proteinaceous materials to attach to clay particles and then improve solid aggregation. When SRM-peptides reach a certain dosage, the active multivalent cations at clay surfaces could be depleted, and the excessive peptides materials started to competitively adsorb to clay surfaces. Considering that the molecular weight of peptides in SRM-peptides varied substantially from 10 kDa to 250 kDa (Figure 1), it was likely that those low-molecular weight peptides with shorter chains were preferentially adsorbed to the clay surfaces in contrast to high-molecular weight ones, leading to a reduced percentage of long-chain peptides adsorption at clay surfaces. As long-chain (higher in molecular weight) molecules are more effective in facilitating inter-particle bridging, an evident decrease in solid aggregation performance was therefore observed in the case of an SRM-peptides overdose. Overall, an optimum SRM-peptides dosage of 14 kg/ton was found to improve the initial settling of SKTs, while the sediment condensation was kept comparable to the untreated d-FFTs. Since the presence of low-molecular weight peptides potentially limited the aggregation performance of SRM-peptides on fine solids, our future work may include the incorporation of certain crosslinking agents to increase the molecular weight of peptides to develop more effective aggregation agents for improved tailings phase separation.

## 4. Conclusions

This research highlights the first study on using peptides derived from specified risk materials (SRM) for oil sands tailings treatment. While less effective than commercial HPAM, SRM-peptides are effective aggregation agents capable of accelerating the settling rate of 4 wt.% kaolinite slurry by 2–3 folds. Similarly, using SRM-peptides at an optimal dosage of 14 kg/ton was found to enable the highest settling rate of the diluted fluid fine tailings, which was ~4 fold higher than the untreated control. The condensation of sediment was found to be subtly affected by the use of SRM-peptides. The released water contained <0.01 wt.% of suspended solids (>0.45 μm), suggesting that it may be possible to send this water back to the oil sands extraction plant for continuous operation.

## Data Availability

Data available upon reasonable request from the authors.
